# Effects of the Novel PFKFB3 Inhibitor KAN0438757 on Colorectal Cancer Cells and Its Systemic Toxicity Evaluation In Vivo

**DOI:** 10.3390/cancers13051011

**Published:** 2021-02-28

**Authors:** Tiago De Oliveira, Tina Goldhardt, Marcus Edelmann, Torben Rogge, Karsten Rauch, Nikola Dobrinov Kyuchukov, Kerstin Menck, Annalen Bleckmann, Joanna Kalucka, Shawez Khan, Jochen Gaedcke, Martin Haubrock, Tim Beissbarth, Hanibal Bohnenberger, Mélanie Planque, Sarah-Maria Fendt, Lutz Ackermann, Michael Ghadimi, Lena-Christin Conradi

**Affiliations:** 1 Clinic of General, Visceral and Pediatric Surgery, University Medical Center Göttingen, Robert-Koch-Straβe 40, 37075 Göttingen, Germany; tiago.deoliveira@med.uni-goettingen.de (T.D.O.); tina.goldhardt@med.uni-goettingen.de (T.G.); marcus.edelmann@stud.uni-goettingen.de (M.E.); nikola.kyuchukov@med.uni-goettingen.de (N.D.K.); jochen.gaedcke@med.uni-goettingen.de (J.G.); mghadimi@med.uni-goettingen.de (M.G.); 2 Institute of Organic and Biomolecular Chemistry, Tammannstraβe 2, 37077 Göttingen, Germany; torben.rogge@chemie.uni-goettingen.de (T.R.); krauch@gwdg.de (K.R.); Lutz.Ackermann@chemie.uni-goettingen.de (L.A.); 3 Clinic of Hematology and Medical Oncology, University Medical Center Göttingen, Robert-Koch-Straße 40, 37075 Göttingen, Germany; Kerstin.Menck@ukmuenster.de (K.M.); annalen.bleckmann@ukmuenster.de (A.B.); 4 Department of Medicine Medical Clinic A, University Hospital Münster, Albert-Schweitzer-Campus 1, 48149 Münster, Germany; 5 Department of Biomedicine, Aarhus University, Høegh-Guldbergs Gade 10, DK-Aarhus C, 8000 Aarhus, Denmark; joanna.kalucka@aias.au.dk; 6 Aarhus Institute of Advanced Studies (AIAS), Aarhus University, 8000 Aarhus, Denmark; 7 National Center for Cancer Immune Therapy (CCIT-DK), Department of Oncology, Copenhagen University Hospital, 2730 Herlev, Denmark; shawez.jmi@gmail.com; 8 Institute of Medical Bioinformatics, University Medical Center Göttingen, Goldschmidtstraße 1, 37077 Göttingen, Germany; martin.haubrock@bioinf.med.uni-goettingen.de (M.H.); tim.beissbarth@bioinf.med.uni-goettingen.de (T.B.); 9 Institute of Pathology, University Medical Center Göttingen, Robert-Koch-Straβe 40, 37075 Göttingen, Germany; hanibal.bohnenberger@med.uni-goettingen.de; 10 Laboratory of Cellular Metabolism and Metabolic Regulation, VIB-KU Leuven Center for Cancer Biology, VIB, Herestraat 49, 3000 Leuven, Belgium; melanie.planque@kuleuven.vib.be (M.P.); sarah-maria.fendt@kuleuven.be (S.-M.F.)

**Keywords:** colon cancer, rectal cancer, glycolysis, KAN0438757, PFKFB3, intestinal organoids

## Abstract

**Simple Summary:**

Glycolysis is one of the hallmarks of cancer. Therefore, the development of novel therapeutical strategies for colorectal cancer targeting glycolysis may improve treatment responses. PFKFB3 expression has been directly associated with enhanced glycolysis, not only in cancer cells but also within the tumor environment. The aim of this study was to evaluate PFKFB3 expression and its correlation with outcome in rectal and colon tumors and to assess the effects of the newly developed PFKFB3 inhibitor KAN0438757 on colorectal cancer cells and intestinal patient-derived organoids. Our results showed that KAN0438757 efficiently targets PFKFB3 expression and was able to affect cancer cell motility, invasion and survival. Additionally, a tumor specific cytotoxic-effect was observed in patient-derived organoids. In vivo, KAN0438757 showed to be well tolerated by mice without systemic toxicity. Our work re-enforces the concept that targeting of glycolysis may be a promising therapeutical approach for colorectal cancer.

**Abstract:**

Background: Despite substantial progress made in the last decades in colorectal cancer (CRC) research, new treatment approaches are still needed to improve patients’ long-term survival. To date, the promising strategy to target tumor angiogenesis metabolically together with a sensitization of CRC to chemo- and/or radiotherapy by PFKFB3 (6-phosphofructo-2-kinase/fructose-2,6-biphosphatase-3) inhibition has never been tested. Therefore, initial evaluation and validation of newly developed compounds such as KAN0438757 and their effects on CRC cells are crucial steps preceding to in vivo preclinical studies, which in turn may consolidate new therapeutic targets. Materials and Methods: The efficiency of KAN0438757 to block PFKFB3 expression and translation in human CRC cells was evaluated by immunoblotting and real-time PCR. Functional in vitro assays assessed the effects of KAN0438757 on cell viability, proliferation, survival, adhesion, migration and invasion. Additionally, we evaluated the effects of KAN0438757 on matched patient-derived normal and tumor organoids and its systemic toxicity in vivo in C57BL6/N mice. Results: High PFKFB3 expression is correlated with a worse survival in CRC patients. KAN0438757 reduces PFKFB3 protein expression without affecting its transcriptional regulation. Additionally, a concentration-dependent anti-proliferative effect was observed. The migration and invasion capacity of cancer cells were significantly reduced, independent of the anti-proliferative effect. When treating colonic patient-derived organoids with KAN0438757 an impressive effect on tumor organoids growth was apparent, surprisingly sparing normal colonic organoids. No high-grade toxicity was observed in vivo. Conclusion: The PFKFB3 inhibitor KAN0438757 significantly reduced CRC cell migration, invasion and survival. Moreover, on patient-derived cancer organoids KAN0438757 showed significant effects on growth, without being overly toxic in normal colon organoids and healthy mice. Our findings strongly encourage further translational studies to evaluate KAN0438757 in CRC therapy.

## 1. Introduction

Colorectal cancer (CRC) is amongst the most important causes of cancer-associated mortality worldwide. Epidemiologically, it is the third most common malignant tumor entity in Western societies, and when epidemiologically studied, the regions of Europe and Oceania share the highest incidence rates [1]. While patients diagnosed at early stages of CRC have a high 5-years survival rate of approximately 82% to 94%, less than 15% of patients with advanced metastatic disease (mCRC) are alive five years after initial diagnosis [1,2]. Besides surgery, chemo- and radiotherapy, specific agents like monoclonal antibodies such as cetuximab, bevacizumab, ramucirumab, aflibercept and others are also used to treat mCRC [3]. Antiangiogenic drugs such as VEGF inhibitors (e.g., bevacizumab) are currently used in the clinic to block tumor vessel formation and therefore are meant to prevent tumor growth and metastasis and thereby improve patients’ survival. Nonetheless a significant fraction of patients shows primary resistance or develops resistance towards antiangiogenic therapy during treatment [4]. Recently, it has been shown that tumor endothelial cells are highly dependent on glycolysis for angiogenesis and survival, and that indirect blockade of glycolysis by administration of the PFKFB3 inhibitor 2E-3-(3-pyridinyl)-1-(4-pyridinyl)-2-propen-1-one (3PO) was able to induce tumor vessel normalization (TVN), normalizing the structure and function of the highly abnormal tumor vessels [5]. Ultimately, this effect led to a reduction in mouse melanoma metastasis due to a reduction of the abnormal vascular permeability/leakiness and improved chemotherapy effectiveness via better intratumoral delivery in tumor-bearing mice [5].

Augmenting the idea of targeting glycolysis by inhibiting the glycolytic activator PFKFB3, recently Gustafsson and colleagues developed a novel PFKFB3 inhibitor compound, KAN0438757 [6]. The authors showed that PFKFB3, beyond its known role for glycolysis [7], is a critical factor for homologous recombination repair of DNA double-strand breaks. Mechanistically, the pharmacological inhibition of PFKFB3 by KAN0438757 impaired the recruitment of ribonucleotide reductase M2 and deoxynucleotide incorporation upon DNA repair, affecting the survival of transformed cells upon radiation-induced DNA damage. These findings suggested that treatment of cancer cells with KAN0438757 induces cellular radio-sensitization and therefore could potentially be used as a new therapeutical approach for cancer treatment.

To date, the strategy of tumor vessel normalization by targeting endothelial cell (EC) metabolism has never been used for the treatment of colorectal cancer patients, and remains untested. The establishment of TVN and tumor radio- and/ or chemo-sensitization in combination with standard therapeutical regimes could potentially improve the therapy response for CRC patients and their long-term outcome. Nevertheless, initial evaluation and validation of KAN0438757 effects on CRC cells are necessary prior to initiating and executing more complex pre-clinical studies. In this study we individually evaluated PFKFB3 expression in normal colon and rectal mucosa and compared it to colon and rectal adenocarcinoma, and correlated its levels with survival rates. Additionally, we assessed the efficiency of KAN0438757 to block PFKFB3 expression in human CRC and endothelial cells and its effects in vitro on cell migration and invasion, cellular viability and cell death. Lastly, we checked the effects of KAN0438757 on colonic patient-derived tumor and normal tissue organoids and its overall toxicity in vivo, in immune-competent C57BL6/N mice, at three different dosages for an interval of 24 h to 21 days. Our findings strongly encourage further translational studies to evaluate the use of KAN0438757 in CRC therapy.

## 2. Results

### 2.1. PFKFB3 Expression in Human CRC Is Associated with Poor Survival

**Figure 1:**
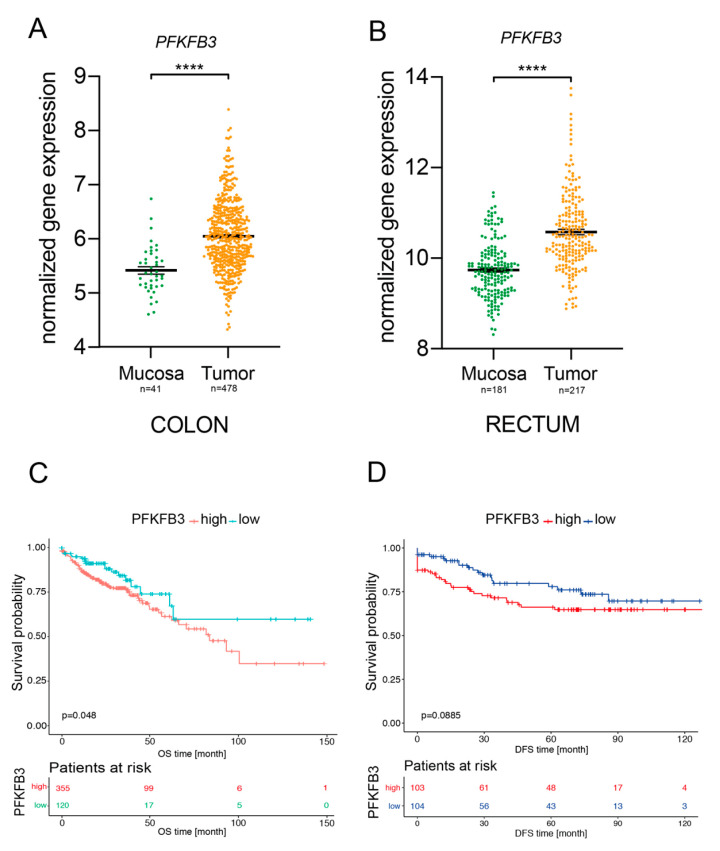
PFKFB3 expression in human colon and rectal cancer. (**A**,**B**) *PFKFB3* mRNA expression on colonic and rectal normal mucosa versus colonic and rectal tumors. (**C**,**D**) Kaplan–Meier plots for colon cancer overall survival (patients *n* = 475), and rectal cancer disease-free survival (patients *n* = 207), respectively, **** *p* < 0.0001.

The importance of glycolysis for the metabolism of gastrointestinal cancers has already been well documented and has been shown to play a relevant role in CRC [8,9,10,11,12]. Upregulation of glycolytic enzymes, metabolite transporters and transcription factors are a well-known phenomenon in different cancers and has been associated with poor prognosis. Moreover, enhanced glycolysis is suggested to induce chemo- and radiotherapy resistance in different cancer cell types [6,12,13,14,15,16,17,18]. In line with this, PFKFB3—a central mediator of glycolysis—has been recognized as an important player within the different aspects of tumorigenesis, not only regulating the survival of tumor cells but also of components within the tumor microenvironment, such as immune and endothelial cells [5,19]. Therefore, we decided to individually evaluate PFKFB3 gene expression in normal colon and rectal mucosa in comparison to colon and rectal adenocarcinoma. Initial comparative analyses from two different large CRC patient cohorts (The Cancer Genomic Atlas Network cohort for colon cancer [20] and a rectal cancer cohort from our clinic, RRID:SCR_007834 oncomine.org) revealed that PFKFB3 mRNA expression is found significantly increased in both colon and rectal adenocarcinoma compared to normal mucosa (Figure 1A,B). We next asked whether PFKFB3 expression would affect disease-free and overall survival rates of colon and rectal cancer patients, respectively. Confirming our hypothesis that increased PFKFB3 expression—and therefore elevated glycolysis—promotes tumorigenesis, Kaplan–Meier plots showed for both colon and rectal cancer and association of enhanced PFKFB3 expression with worse survival rates (*p* = 0.04 for colon cancer and *p* = 0.08 for rectal cancer) (Figure 1C,D). In sum, these data show that PFKFB3 expression is found enhanced in colorectal cancer and its expression inversely correlates with the survival of colon and in trend of rectal cancer patients.

### 2.2. KAN0438757 Efficiently Reduces PFKFB3 Expression in Colorectal Cancer Cells, without Reducing Its Transcriptional Regulation

**Figure 2:**
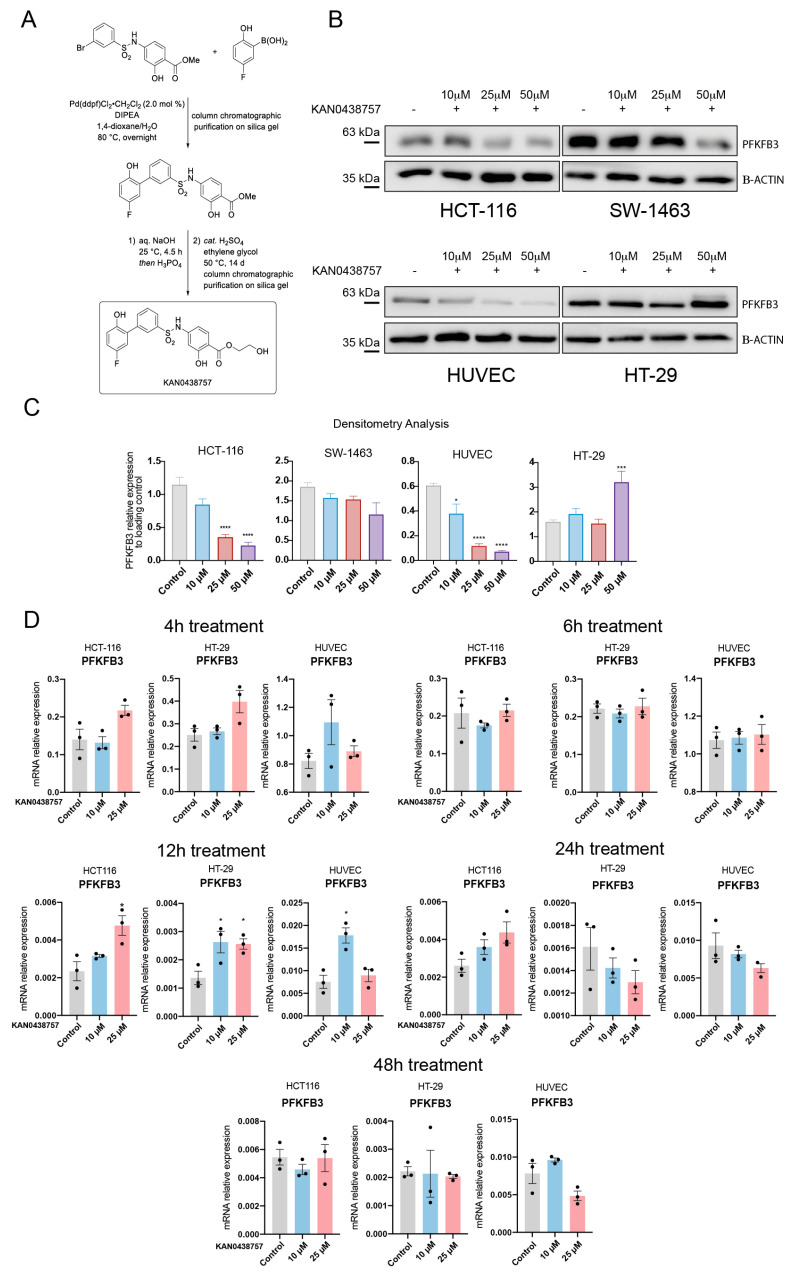
KAN0438757 effects on PFKFB3 expression in cancer cells and HUVECs. (**A**) KAN0438757 synthetization protocol steps. (**B**) Immunoblot. PFKFB3 inhibition in colorectal cancer cells and HUVECs upon 12 h treatment with KAN0438757. (**C**) Immunoblot relative quantification by densitometry (relative to B-ACTIN loading control). (**D**) RT-PCR analysis. Relative mRNA expression of *PFKFB3* in HCT-116, HT-29 and HUVEC cells upon 4, 12, 24 and 48 h treatment with KAN0438757. Data is ± SEM. * *p* < 0.05, *** *p* < 0.001, **** *p* < 0.0001.

To evaluate whether the newly developed PFKFB3 inhibitor [6] (Figure 2A) would be able to reduce PFKFB3 protein expression we decided to treat human umbilical vein endothelial cells (HUVEC) and the CRC cell lines HCT-116, HT-29 and SW-1463 in vitro for 12 h with three different concentrations (10, 25 and 50 μM) of KAN0438757. Immunoblot analysis showed that treatment with KAN0438757 was able to effectively reduce PFKFB3 expression in HCT-116, SW-1463 and HUVECs in a concentration-depended manner (Figure 2B,C). Moreover, HCT-116 cells were shown to be more susceptible to PFKFB3 inhibition by KAN0438757 than SW-1463 cancer cells (Figure 2B,C), whilst HT-29 cells interestingly upregulated PFKFB3 expression upon 50 μM of KAN0438757. Next, to evaluate whether modifications in PFKFB3 protein expression were due to possible changes in its transcription, we incubated HCT-116 and HT-29 cancer cells for 4, 6, 12, 24 and 48 h with 10 and 25 μM KAN0438757 and subsequently performed RT-PCR analyses for PFKFB3 mRNA expression (Figure 2D). In addition to this set up, we also analyzed the PFKFB3 mRNA levels in primary, lower passage HUVECs, as it has been shown that these cells have a strong glycolytic metabolism and therefore transcriptional changes for PFKFB3 could be easily detected [7,21]. RT-PCR analysis of KAN0438757-treated HCT-116 and HUVECs did not present any consistent reduction in PFKFB3 mRNA levels. Interestingly, PFKFB3 mRNA levels even showed to be significatively enhanced in HT-29 cells at the 12 h time point, in both 10 and 25 μM concentrations, although this was not detected by our immunoblot analysis (Figure 2B,C). Taken together, these results show that KAN0438757 can inhibit PFKFB3 protein expression in HUVEC and CRC cells and suggests that this inhibition might be independent of reduced PFKFB3 mRNA transcription levels.

### 2.3. KAN0438757 Affects the Energy Metabolism of HUVECs and Colorectal Cancer Cells

**Figure 3:**
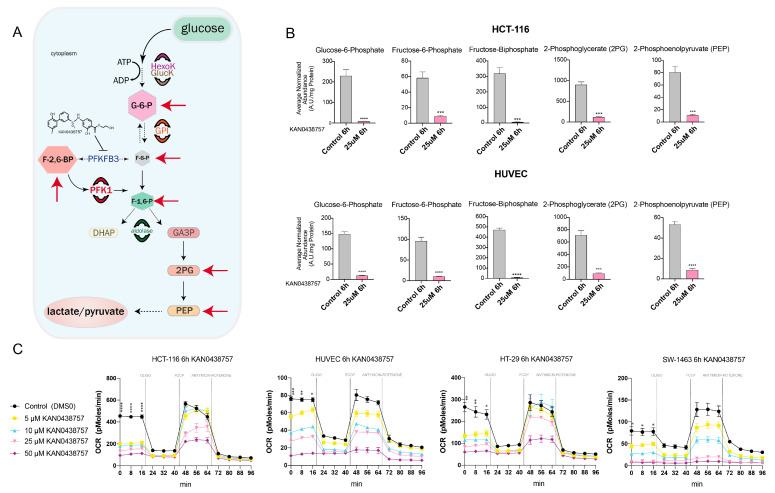
KAN0438757 effectively reduces glycolytic metabolites’ levels. (**A**) Glycolysis schematic representation. Red arrows mark the metabolites directly affected by KAN0438757 treatment in vitro. (**B**) LC-MS metabolomic analysis for HCT-116 cancer cells and HUVECs after 6h treatment with KAN0438757. (**C**) Seahorse analysis for oxygen consumption rate (OCR) evaluation performed with colorectal cancer cells and HUVECS upon 6 h treatment with KAN0438757. (**C**) Data is representative of three independent experiments. Data is ± SEM. * *p* < 0.05, ** *p* < 0.01 *** *p* < 0.001, **** *p* < 0.0001.

The key steps of the glycolytic pathway and its metabolic products are well known, starting with the glucose uptake and terminating with the production of lactate [22,23]. The phosphofructo-1-kinase (PFK-1)-mediated phosphorylation is an initial and crucial step for the setting of several chemical reactions, which will result in the generation of innumerous metabolites that in turn feed forward the regulation of glycolysis (Figure 3A). The production of fructose 2,6- and 1,6-biphosphate (F-2,6-BP and F-1,6-BP), as well as of 2-phosphoglycerate (2PG) and phosphoenolpyruvate (PEP) are direct products of this metabolic flux [24]. Therefore, using LC-MS we evaluated the levels of those glycolytic metabolites in HUVECs and HCT-116 cells, after 6 and 24 h of 25 μM KAN0438757 treatment. Strikingly, a strong reduction of the metabolites glucose-6-phosphate, fructose-6-phosphate, fructose-biphosphate (combined F-2,6-BP and F-1,6-BP), 2PG and PEP was observed at both time points (Figure 3B and Figure S1A). Next, to further evaluate whether the reduction of glycolytic metabolites by KAN0438757 was also associated with effects on mitochondrial respiration and changes in basal oxidative phosphorylation, we have subjected HUVECs and colorectal cancer cells to a 6 h treatment with KAN0438757 and measured their oxygen consumption rate (OCR) by Seahorse^TM^ analysis. OCR was measured under basal conditions and after the addition of the inhibitors oligomycin, trifluoromethoxy carbonylcyanide phenylhydrazone (FCCP), antimycin and rotenone. Corroborating our metabolomics findings, all four tested cell lines showed reduced baseline cellular ORC starting at 5 μM of KAN0438757 (Figure 3C, Figure S3). Thus, these results evidence the ability of the PFKFB3 inhibitor KAN0438757 to reduce glycolysis in normal endothelial and CRC cells.

### 2.4. PFKFB3 Inhibition by KAN0438757 Affects Cellular Growth, Reduces Cell Viability and Induces Cancer Cell Death in a Concentration-Dependent Manner

**Figure 4:**
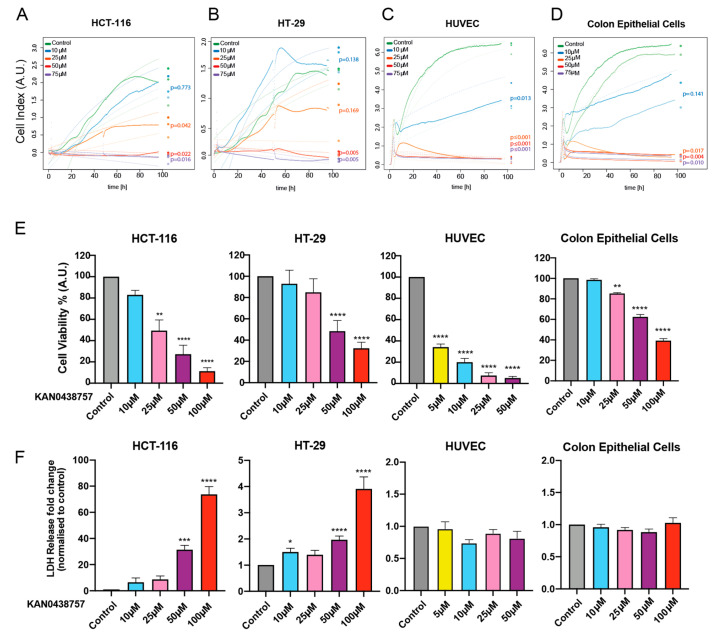
PFKFB3 inhibition by KAN0438757 reduces cancer cell proliferation and induces cell death in a concentration-dependent manner. (**A**–**D**) xCELLigence assay performed with HCT-116 and HT-29 colorectal cancer cells, HUVECs and normal colon epithelial cells during 96 h under different concentrations of KAN0438757 (continuous line); the dotted lines represent the fitted saturation curve in logarithm. (**E**) CellTiterBlue assay performed with HCT-116, HT-29, HUVECs and normal colon epithelial cells during 48 h. (**F**) LDH assay performed with HCT-116, HT-29, HUVECs and normal colon epithelial during 48 h. Data is ± SEM. (**A**–**F**) Combined data from three independent experiments. * *p* < 0.05; ** *p* < 0.01; *** *p* < 0.001; **** *p* < 0.0001.

To evaluate whether inhibition of glycolysis by KAN0438757 would affect the cellular morphology and growth of normal colon epithelial cells (CRL-1831), colon cancer cells or HUVECs we performed an xCELLigence assay [25,26]. The real-time cell analysis executed by the xCELLigence assay provides a quantitative readout of cellular size and shape, cell proliferation and cell-substrate attachment, which allowed us to monitor the cells’ electrical impedance for a period of 96 h and to evaluate their morphology and proliferative capacity under different concentrations of KAN0438757. As shown in Figure 4A,B, HCT-116 and HT-29 cancer cells were able to keep their morphological characteristics (cell size, cell-substrate attachment quality) and proliferative capacity at concentrations of 10 and 25 μM KAN0438757. Higher concentrations of KAN0438757 (50 and 75 μM) significantly abrogated cancer cell proliferation and affected cell-substrate attachment quality (*p* < 0.05). Expectedly, HUVECs were highly susceptible to PFKFB3 inhibition and the parameters measured were already affected at 10 μM KAN0438757 (Figure 4C). Interestingly, normal colon epithelial cells were impaired only with concentrations ≥25 μM KAN0438757 (Figure 4D). Next, to further validate the xCELLigence results and to directly evaluate cellular viability and cell death upon inhibition of glycolysis by KAN0438757 in normal, non-transformed and colorectal cancer cells, we subjected HCT-116, HT-29, HUVECs and normal colon epithelial cells to both a CellTiterBlue (viability) and a lactate dehydrogenase (LDH) release (cell death) assay. Corroborating our previous findings, after 48 h we observed that KAN0438757 was able to affect the ability of cells to convert resazurin into its product resorufin, showing a clear effect on the cellular metabolic capacity and viability (Figure 4E). Additionally, we found that higher concentrations of KAN0438757 (50 and 100 μM) were able to significantly increase cell death rates in HCT-116 and HT-29 cancer cells after 48 h treatment (Figure 4F). Intriguingly, although exhibiting intrinsically higher levels of LDH release when compared with CRC cells, HUVECs and normal colon epithelial cells showed enhanced resistance to KAN0438757-induced cell death, as LDH release levels were not significantly altered even with a concentration of 50 μM KAN0438757 (Figure 4F), an intriguing finding that instigated us to further evaluate KAN0438757 effects on cell death in a more complex in vitro system to circumvent possible assay limitations. In sum, these findings suggest that KAN0438757 is able to robustly reduce colon cancer cells viability in a concentration-dependent manner.

### 2.5. KAN0438757 Treatment Selectively Affects Intestinal Patient-Derived Tumor Organoids’ Morphology and Growth Preserving Normal Colon Organoids

**Figure 5:**
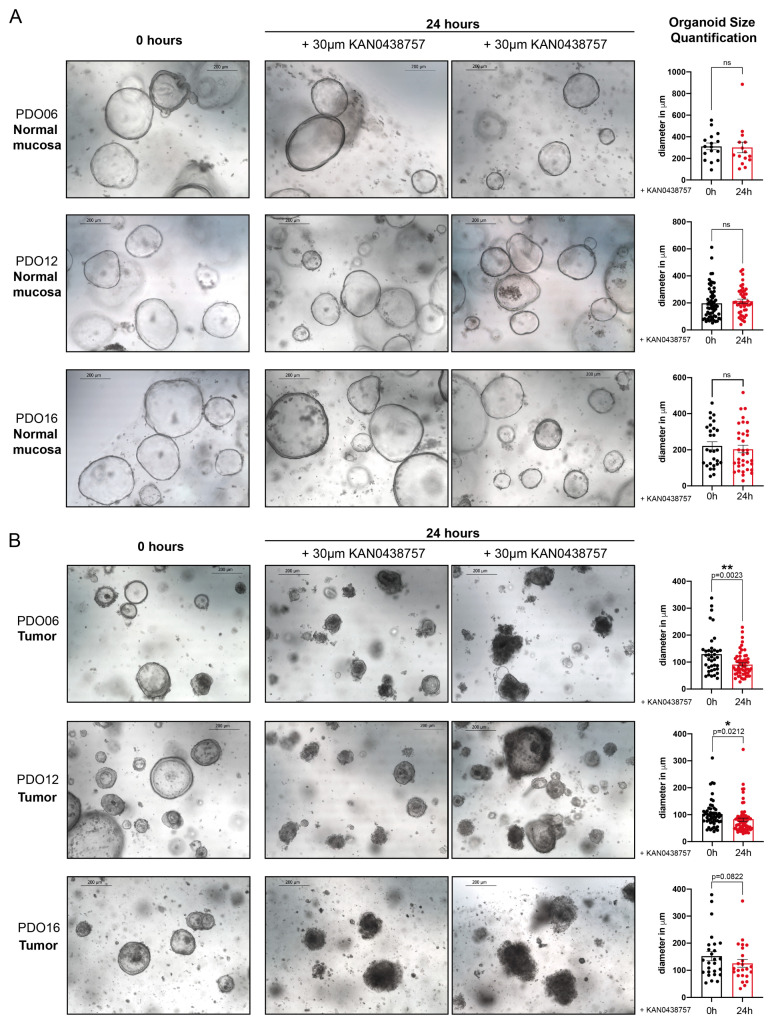
KAN0438757 effects on intestinal patient-derived organoids. (**A**,**B**) Representative pictures of normal colon (mucosa) and tumor patient-derived organoids, treated with 30 μM KAN0438757 for 24 h and their size quantification (right-side panel). Scale bars, 200 μm. Data is ± SEM. * *p* < 0.05; ** *p* < 0.01; ns, *p* > 0.05.

Lately, the use of patient-derived organoids (PDOs), has been consistently increasing, as they have shown to be a robust and relevant pre-clinical model [27]. In addition to its clinical relevance, the full understanding of the molecular mechanisms involved in CRC is crucial for the development of new therapeutical targets and it can only be achieved through pre-clinical models that accurately recapitulate the heterogeneity of the disease [27,28,29,30,31]. Therefore, our findings that KAN0438757 could reduce cancer cell viability while preserving normal, non-transformed cells prompted us to further investigate its effects in a more relevant and complex in vitro system. Consequently, we decided to establish and treat three different right-sided intestinal patient-derived tumor organoids and their matched normal mucosa with 30 μM of KAN0438757 and evaluated its effects on organoid morphology and growth. Impressing, after 24 h we could observe that whilst normal mucosa organoids maintained their morphology without significant signs of cellular toxicity (Figure 5A), tumor organoids’ morphology was strongly affected, with organoid disintegration and an increased number of surrounding single-cells, a clear sign of cell death-induction and reduced organoid viability [32,33] (Figure 5B). Moreover, a significant reduction in size in the remaining organoids could be observed in two of the three analyzed PDOs (Figure 5A,B). These findings further supported our previous results that cancer cells could be selectively targeted by KAN0438757 sparing normal, non-transformed cells from immediate severe harm.

### 2.6. Cancer Cell Motility and Invasiveness Are Reduced by KAN0438757

**Figure 6:**
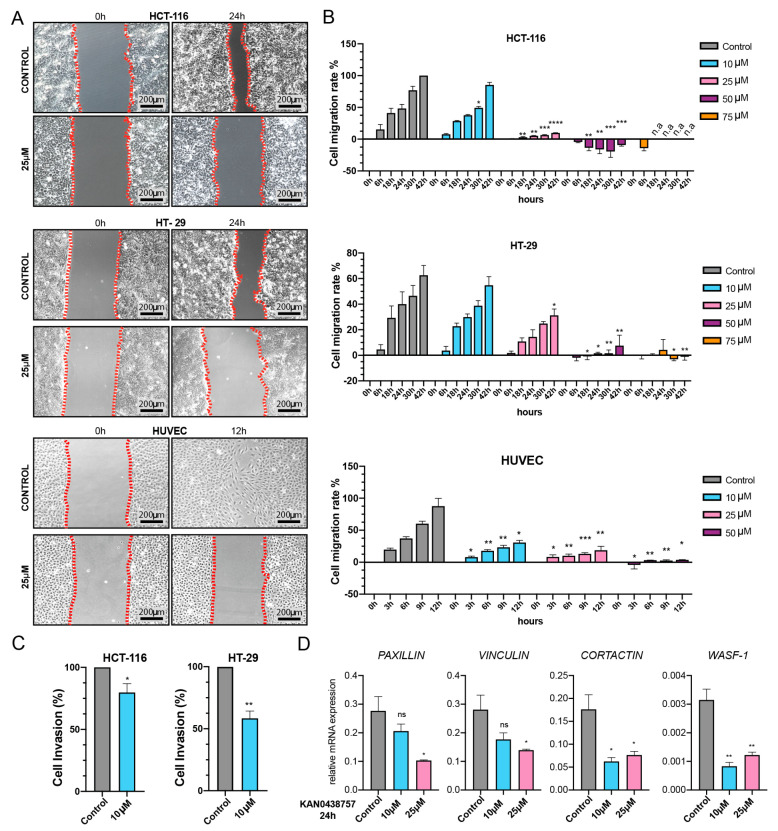
PFKFB3 inhibition by KAN0438757 alters cell motility and cancer cell invasion capabilities. (**A**) Representative pictures of migration assay performed with 25 μM KAN0438757. (**B**) Migration assay quantification performed with HCT-116, HT-29 and HUVEC cells under different concentrations of KAN0438757 for 42 h (cancer cells) and 12 h HUVEC cells. (**C**) Boyden chamber invasion assay quantification performed with HCT-116 and HT-29 cancer cells for 96 h. (**D**) RT-PCR analysis for cell migration-and cytoskeleton-associated genes in HUVECs. Data is ± SEM. (**B**,**C**) combined data from three independent experiments. (**D**) data is representative of three independent experiments. * *p* < 0.05; ** *p* < 0.01; *** *p* < 0.001; **** *p* < 0.0001; ns, *p* > 0.05.

The results obtained by the xCELLigence assay suggested that PFKFB3 inhibition by KAN0438757 could affect cell-substrate attachment (Figure 4A–D), thus we decided to assess the effects of KAN0438757 on cell motility and cancer cell invasiveness in vitro. Therefore, we first performed a migration assay with HCT-116, HT-29 and HUVECs under different inhibitor concentrations for up to 42 h. Interestingly, KAN0438757 administration was able to reduce migration capabilities of all three tested cell lines. HCT-116 cancer cells and HUVECs were significantly affected by KAN0438757 at starting concentrations of 10 μM, whilst effects on the migration of HT-29 were observed at 25 μM KAN0438757 (Figure 6A,B). Subsequently, we set out to determine whether cancer cell invasiveness would also be affected by KAN0438757. For this reason, we performed an invasion assay with HCT-116 and HT-29 cells using a low concentration of KAN0438757 (10 μM), which previously showed no major effects on cell viability (Figure 4E). After 96 h, quantification of the invaded cells showed that 10 μM KAN0438757 was able to significantly reduce cancer cell invasiveness in both cell lines (Figure 6C). Corroborating these findings, additional RT-PCR analysis of HUVECs treated with KAN0438757 for 12, 24 and 48 h evidenced reduced expression of the cell migration- and invasion-associated genes *PAXILLIN*, *VINCULIN*, *CORTACTIN* and Wiskott-Aldrich syndrome protein 1 (*WASF-1*) (Figure 6D, Figures S1B and S4A–D). Intriguingly, HCT-116 and HT-29 cancer cell lines did not show the same relevant reduction, and even showed increased expression of some of those genes (Figure S2A,B).

### 2.7. In Vivo Administration of KAN0438757 Shows No Relevant Systemic Toxic Effects

**Figure 7:**
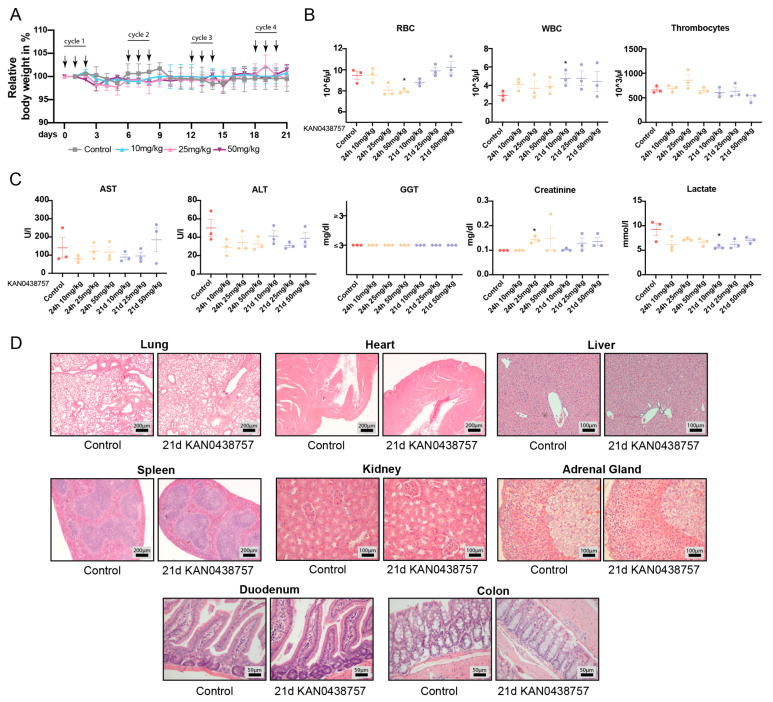
In vivo toxicity testing of KAN0438757 in immune-competent mice. (**A**) Relative weight curves of C57BL6/N mice treatment with 10 mg/kg (*n* = 3), 25mg/kg (*n* = 3) and 50 mg/kg (*n* = 3) KAN0438757 (arrows, i.p. injections). Control, DMSO (*n* = 3). (**B**,**C**) Whole blood counts (RBC, WBC, thrombocytes) and serum biochemical evaluation of aspartate transaminase (AST), alanine transaminase (ALT), gamma-glutamyl transferase (GGT), creatinine and lactate after treatment periods of 24 h to 21 days with KAN0438757. (**D**) Representative pictures of histological analysis (H&E) of lungs, heart, liver, spleen, kidneys, adrenal glands, duodenum and colon from mice treated 21 days (21 d) with KAN0438757 (25 mg/kg dosage). * *p* < 0.05; Data is ± SEM.

The toxicity testing of newly developed compounds is crucial for the establishment of new promising therapeutic targets. The evaluation of drug toxicity on biological systems can reveal important tissue- organ- and dose-specific effects of the investigated compound [34]. Therefore, based on the previous results obtained with CRC cells and PDOs we decided to assess the possible toxic effects of the new PFKFB3 inhibitor KAN0438757 in vivo. For this purpose, we subjected nine weeks-old, immune-competent mice (C57BL6/N) to three different dosages (i.p., 10; 25; 50 mg/kg, which were chosen based in our previous experience with PFKFB3 inhibitors [5,35]) for an interval of 24 h to 21 days. Acute toxicity testing (24 h) was carried out to determine the immediate systemic effects of a single dose of KAN0438757. Prolonged 21 days testing was carried out to evaluate its systemic effects on at least four consecutive treatment cycles of KAN0438757 (one cycle = 3 successive i.p. injections of KAN0438757 followed by 3 days break) (Figure 7A), simulating its possible future use in exploratory, pre-clinical animal studies and future clinical use. During the treatment, animal groups from all three different dosages did not show any visible clinical alterations or changes in behavior or food/drink intake. Additionally, regular weight measurements did not show any significant weight losses or gains in both tested intervals (Figure 7A). To further evaluate the systemic effects of KAN0438757, we investigated the blood counts and the levels of hepatic function markers of mice treated with the inhibitor for 24 h to 21 days. Surprisingly, even in the highest dose of 50 mg/kg, no relevant changes in all tested parameters were detected (Figure 7B,C). Lastly, to rule out possible toxic effects of KAN0438757 in vivo on different organs, we histologically analyzed H&E stainings from all treated mice. Corroborating our previous findings, histological analysis of lungs, heart, liver, spleen, kidneys, adrenal glands, duodenum and colon tissues did not reveal any relevant morphological alterations (Figure 7D). Thus, these results indicate that the in vivo administration of KAN0438757 in mice is well tolerated and does not trigger major systemic or toxic alterations.

## 3. Discussions

The validation of newly developed compounds, many of which may have relevant clinical activity, and the detailed identification of their specificity and possible off-target effects are crucial steps for the establishment of novel therapeutical approaches in oncology [36,37]. Enhanced glycolysis, metabolic reprograming and neoangiogenesis are established hallmarks of cancer and therefore their therapeutic targeting is of great interest in order to advance treatment approaches [38]. Recently, PFKFB3 has been shown to be a promising target, not only due to its crucial involvement in glycolysis, but also because of its ability to induce tumor vessel normalization and to lead to drug sensitization [5,6,39,40]. Knowing that a tumor-promoting metabolic reprograming is found in several malignancies, including CRC [41], it is imperative that PFKFB3 inhibition should be studied further. Interestingly, although being both originating from the large intestine, colon and rectal tumors are considered to be very distinct in their molecular and clinical characteristics. They do not differ only in respect to their anatomic localization and proximity to the anocutaneous line but they also have recognized differences in their pathogenesis, surgical approaches, molecular profile and multimodal treatment [4,39]. The finding that PFKFB3 expression is significantly increased in both colon and rectal carcinoma confirms our hypothesis that a metabolic shift towards glycolysis happens during colon- and rectal-carcinogenesis, and that indeed its increased expression affects patients’ outcomes.

To comparatively represent this situation in vitro, we decided to use cancer cell lines, which also differ in their origin, as HCT-116 and HT-29 cells are derived from colon cancer and SW-1463 cells are from rectal cancer [42]. To evaluate the effects on endothelial cells, which are extremely depended on glycolysis and represent relevant targets for future anti-angiogenic therapies we used primary HUVECs. Interestingly, all the used cancer cells presented intrinsic differences in their response to a PFKFB3 blockade upon KAN0438757 treatment. These differences were clearly depicted by, e.g., their variable PFKFB3 protein levels (Figure 2B,C) and by their mRNA expression levels for migration-related genes (Figure 6D and Figure S2A), upon treatment with KAN0438757. We have considered that their distinct mutational status could be responsible for the observed differences. HCT-116 is highly hypermutated and microsatellite instable (MSI), whilst HT-29 and SW-1463 are non-hypermutated, microsatellite stable (MSS) cell lines [43,44,45,46,47,48]. Furthermore, the cancer cell lines differ in KRAS, BRAF and PIK3CA mutations, which are known to directly affect metabolic reprograming through their involvement in the MAPK/ERK signaling pathway [46,49,50]. HT-29 and HCT-116 cells also differ in their EGFR expression, which is involved in cancer cells migration and invasion capabilities [44]. However, whether HCT-116, HT-29 and SW-1463 present cell-specific differences in PFKFB3 regulation upon glycolysis disruption remains to be further evaluated.

Cancer cells are well-known for their metabolic reprograming and for the use of glycolysis as a main energy source, however in most mammal cells adenosine triphosphate (ATP) is generated by oxidative phosphorylation, which takes place in the mitochondria [51]. The detection that KAN0438757 not only effectively reduced glycolytic metabolites’ levels but also could interfere with cancer cells oxidative phosphorylation, presumable due to a reduction in pyruvate availability (Figure 3A–C) is of great interest for further metabolic studies in oncology. Our findings that the new PFKFB3 inhibitor was able to robustly affect HCT-116 and HT-29 cancer cells viability in a concentration-dependent manner, without profound effects on induced-cell death in HUVECs and normal colon epithelial cells are in accordance with what was previous suggested by Gustafsson and colleagues [6] that normal, non-transformed and less proliferating cells would present higher tolerability to KAN0438757 at concentrations which can disrupt cancer cell survival. Nevertheless, to circumvent possible limitations and/or misleading results unforeseen by us from the LDH assay with normal, non-transformed cells (HUVECs and colon epithelial cells), as such as undetected early induced death (occurring outside the experimental time point evaluated by us) or by eventual LDH degradation in the media of these cells, we decided to assess whether normal colon mucosal organoids would tolerate KAN0438757 treatment. The striking results obtained with intestinal PDOs further supported the idea that KAN0438757 could possibly target tumor cells preserving normal tissues, a very desired scenario in oncological therapeutical approaches, where severe systemic toxicity affects patients’ outcomes [52,53,54,55,56].

Our findings are corroborated by other studies, which have also shown that PFKFB3 expression is involved in cancer cell viability and that PFKFB3 inhibition can induce cell death [57,58,59,60].

Previous studies have already shown that the inhibition of glycolysis in cancer cells was accompanied by cytoskeletal rearrangements [61,62,63]. The observation that PFKFB3 inhibition by KAN0438757 reduces cell migration and invasion capabilities in both transformed and non-transformed cells is supported by those studies. The clear reduction of the expression of migration- and invasion-related genes *PAXILLIN*, *VINCULIN*, *CORTACTIN* and *WASF-*1 upon KAN0438757 treatment was observed exclusively in HUVECs, suggesting that (i) in cancer cells other molecular mechanisms might be involved in their diminished motility capabilities (ii) or/and unknown compensatory mechanisms triggered by KAN0438757 treatment. Regarding the latter, it is possible that the inhibition of glycolysis through KAN0438757 might trigger changes in the levels of the TCA cycle intermediates and aminoamides, which in turn are known to be able to support epigenetic compensatory alterations [64,65]. Nevertheless, those open questions need to be further addressed in complementary studies.

Lastly, according to Sun Hongmao [66], unexpected drug toxicity is one of the main causes for failures of otherwise promising drug candidates and for their withdrawal from pharmaceutical pipelines. Our main goal with the early in vivo testing of KAN0438757 was to assess its possible systemic toxicity and prioritize its future pre-clinical evaluation, as in vivo pre-clinical models are currently the gold standard for assessing drug safety and human risk [67]. Thus, the finding that mice can tolerate a continuous KAN0438757 administration for longer periods (21 days), even at higher doses (50 mg/kg) without any signs of high-grade toxicity strongly encourage further translational studies to evaluate it in CRC therapy.

## 4. Materials and Methods

### 4.1. Cell Culture

All cell lines, with exception of HUVECs, were from the American Type Culture Collection (ATCC, Manassas, VA, USA). HCT-116 and HT-29 were kept at 37 °C, humidified 5% CO_2_ incubators and cultured with McCoys medium supplemented with 10% fetal bovine serum (FBS) (Biochrom, Berlin, Germany) and 1% Pen/Strep (Gibco, Thermo Fischer Scientific, Waltham, MA, USA). SW-1463 cells were kept at 37 °C, humidified atmospheric-air incubators and cultured with Leibovitz’s L-15 medium supplemented with 10% FBS (Biochrom) and 1% Pen/Strep (Gibco). Primary, single-donor early passage HUVECs were kindly provided by Dr. Joanna Kalucka (Department of Biomedicine, Aarhus University, Aarhus, Denmark) and cultured at 37 °C, 5% CO_2_ humidified incubators with Endopan 300SL medium (PAN Biotech, Aidenbach, Germany). Cells between passages five to twelve (HCT-116, HT-29, SW-1463) and passages two to three (HUVECs) were used for experiments. Normal colon epithelial cells (passages 1 and 2) were cultures with DMEM/F12 medium (ATCC) supplemented with 10% non-inactivated FBS (Biochrom), 10 ng/mL cholera toxin (Sigma), 0.005 mg/mL insulin (Gibco), 0.005 mg/mL transferrin (Gibco), 100 ng/mL hydrocortisone (Sigma) and 20 ng/mL human EGF (Gibco). The final DMSO concentration experienced by cells in vitro was ≤ 0.25%.

### 4.2. Patient-Derived Organoids

Fresh tissue samples used in this study were provided by the University Medical Center Göttingen (UMG), Germany. A written informed consent was obtained from all patients, and the study was approved by the Ethical Committee of the University Medical Center Göttingen (UMG Antragsnr. 25/3/17, from 2017). Intestinal patient-derived organoids were established and cultured according to Sato et al. 2011 [33] and Mihara et al. 2016 [68], from three different right-sided colon tumors and their adjacent non-pathological (normal) mucosa, after surgical tumor resection. Briefly, human normal organoids were cultured in “complete medium” composed of advanced DMEM/F12 supplemented with 1 × Glutamax, 10 mM Hepes, 1 × penicillin/streptomycin (10,000U) (all from Gibco), 10 mM nicotinamide (Sigma), 1 × B27 (Gibco), 500 nM A83-01 (Tocris), 10 µM SB202190 (MedChem Express, Monmouth Junction, NJ, USA), 1.25 mM *N*-acetylcysteine (Sigma), 20% R-spondin conditioned media (CM) (in house made), 50% Wnt3a CM [68] (in house made), 10% Noggin CM (in house made) and 50 ng/mL human EGF (Gibco). 10 µM Y-27632 (Adooq Biosciences, Irvine, CA, USA) was added to the medium after organoid extraction and seeding. 100 µg/mL Primocin (InvivoGen, Toulouse, France) was added to the medium only during extraction and organoid’s first passage. The phenotype of all normal organoids was confirmed by growth arrest in medium lacking Wnt3a CM. Tumor organoids were cultured in “complete medium” containing extra 1 × N2 supplement (Gibco) and lacking Wnt3a CM. Experiments with PDO06 (colon ascendens), PDO12 (coecum) and PDO16 (ascendens, right-colic flexure) were all performed at their second passage, after organoids were well formed (normal mucosa organoids took approx. 4–5 days to grow, tumor organoids between 7–10 days). Organoids’ “complete medium” was supplemented with 30 μM KAN0438757 and its effects were evaluated after 24 h by microscopical images, using a Leica High Speed EC3 camera (Leica, Wetzlar, Germany) on a Leica DM IL inverted microscope (Leica). Additional organoid controls were treated with drug diluent (0.20% *v*/*v* DMSO). Size quantification was performed using ImageJ 1.43u Software (National Institutes of Health, Bethesda, MD, USA).

### 4.3. CellTiterBlue Assay

The assay was performed according to the manufacturer’s protocol (Promega, Madison, WI, USA). Briefly, 1 × 10^4^ (HCT-116, HT-29) or 1.5 × 10^4^ (HUVECs and normal colon epithelial cells) cells were seeded into 96-well, black-walled tissue culture plates (Corning, Corning, NY, USA). After 24 h incubation at 37 °C, 5% CO_2_ the medium was replaced by 100 µL medium containing KAN0438757. One hour before the selected time point 10 µL of Resazurin, was added to each well. Reduced Resazurin (Resarufin) was measured using the Victor™ Multilabel Plate Reader (PerkinElmer, Inc., Waltham, MA, USA) at 560_ex_/590_em_ nm wavelength. Every condition was tested in triplicates and the assay was performed three times.

### 4.4. LDH-Assay

KAN0438757-induced cell death was evaluated using the LDH Cytotoxicity Detection Kit-PLUS (Roche, Diagnostics, Mannheim, Germany), following the manufacturer’s protocol. Approximately 7.5 × 10^3^ (HCT-116, HT-29) or 1.5 × 10^4^ (HUVECS and normal colon epithelial cells) cells were seeded into 96-well-plates (Corning, Corning, NY, USA). After 24 h incubation the medium was replaced by 100 µL medium containing KAN0438757 at different concentrations. Fifteen minutes before the end of the experiment 5 µL lyse-solution was added into positive-high control wells. Next, 100 µL of reaction-solution was added in each well and further incubated at room temperature in the dark for 30 min. Lastly, 50 µL stop-Solution was added in each well and the absorbance of Formazan salt was read using the Victor™ Multilabel Plate Reader spectrophotometer (PerkinElmer, Inc., Waltham, MA, USA) at 490 nm filter. Every condition was tested in triplicates and the assay was performed three times.

### 4.5. Invasion Assay

The invasion assay was performed as previously described [69]. Essentially, 1 × 10^5^ cells were seeded into the upper well of the boyden chamber in 1000 µL medium. KAN0438757 was added in different concentrations into both sides of the chamber. After 96h incubation at 37 °C, 5% CO_2_ the medium from the upper chamber was removed and the content of the lower part, containing the invaded cells (floating as well as adherent cells), was collected for each condition. Next, cells were centrifuged at 500 g, for 5 min, stained with 0.4% Trypan Blue (Carl Roth, Karlsruhe, Germany) and manually counted under the microscope using a Neubauer-improved Chamber (Paul Marienfeld GmbH, Lauda-Königshofen, Germany).

### 4.6. xCELLigence Assay

Cells (1 × 10^4^/100 µL) were seeded in 16-well-plates, bottom-coated with golden microelectrodes and incubated in the xCELLigence Real-Time Cell Analysis (RTCA) S16 instrument (both, ACEA Bioscience, San Diego, CA, USA) at 37 °C, 5%CO_2_ conditions. The instrument constantly measures the cellular impedance, which was recorded in arbitrary units (A.U.) every 5 min for 96 h. Inhibitors were added directly in the cell suspension after the first readout and replenished after 48 h incubation. Recorded data was analyzed and the cell index generated using the RTCA Software Lite version 1.2 (ACEA Bioscience). Briefly, the cell index is generated based on changes in impedance, which is influenced by modifications in cell-proliferation, cell-adhesion, morphology and shape [25,26]. Every condition was tested in triplicates and the assay was performed three times. Conditions were compared by fitting a saturation curve in logarithm and predicting the 80 h time point which was then compared using a *t*-test.

### 4.7. Animal Experiments

C57BL6/N mice were from the Jackson Laboratory (Bar Harbor, ME, USA). Animals were housed at the UBFT animal facility from the University Medical Center Göttingen, under specific pathogen-free conditions and controlled 12 h light/dark cycle. Females, nine weeks-old were used in the experiments. Food and water were provided ad libitum. To evaluated the short- and long-term toxicity of KAN0438757 in vivo, mice received different dosages of the inhibitor (10 mg/kg, 25 mg/kg, 50 mg/kg) in intervals of 24 h to 21 days. The drug was diluted in a solution of 50% *v*/*v* DMSO/NaCl 0.9%. For long-term toxicity evaluation (21 days), mice were injected every three days, once a day i.p., for 3 consecutive days with KAN0438757. Control mice received only the 50% *v*/*v* DMSO/NaCl 0.9% diluent. Blood counting was performed using the CELL DYN Sapphire Hematology Analyzer (Abbott, Green Oaks, IL, USA). AST, ALT, GGT, Creatinine and Lactate levels were evaluated using the Architect Plus C16000 Analyzer (Abbott) with the following kits: AST (Activated Aspartate Aminotrasferase, ref. 8L91-21, Abbott); ALT (Activated Alanine Aminotrasferase, ref. 8L92-21, Abbott); GGT (Total Bilirubin, ref. 7P32-21, Abbott); Creatinine (Creatinin PAP FS, DiaSys Diagnostic Systems GmbH, Holzheim, Germany); Lactate (Lactat, DiaSys Diagnostic Systems GmbH). All procedures were approved by the Niedersächsisches Landesamt für Verbraucherschutz und Lebensmittelsicherheit (LAVES, TVA 33.9-45502-04-20/3490), Germany.

### 4.8. Seahorse Analysis

Seahorse analysis was performed as previously described [70], with minor modifications. Cells were seeded into 96-well plates (Aligent Technologies, Santa Clara, CA, USA) and incubated overnight. Next day, complete medium was exchanged for serum-free medium containing different concentrations of KAN0438757 for 6 h before analysis at the Seahorse XF instrument (Aligent). All injected reagents (oligomycin, FCCP, actinomycin and Rotenone) were from Sigma.

### 4.9. Histological Analysis

Harvested organs were fixed overnight in 4% PFA (Electron Microscope Sciences, Hatfield, UK) at 4 °C. The following day, samples were transferred to 70% ethanol and processed by Leica TP 1020 Tissue Processor (Leica, Wetzlar, Germany), according to the manufacturer’s protocol. After dehydration tissues were embedded in paraffin blocks and stored at room temperature (RT). For hematoxylin and eosin staining (both Carl Roth), 2-4 μm tissue sections fixed on Super Frost glass slides (Thermo Fischer Scientific) were treated as previously described [71].

### 4.10. Immunoblot Analysis

Cells were lysed with 50 mM Tris-HCl (pH 7.5), 250 mM NaCl, 30 mM EDTA, 30 mM EGTA, 25 mM sodium pyrophosphate, 1% Triton-X 100, 0.5% NP40, 10% Glycerol, 1 mM DTT (all from Sigma-Aldrich, Saint Louis, MO, USA) supplemented with EDTA-free mini protease inhibitor cocktail and PhosSTOP tablets (both, Roche Diagnostics). Pierce BCA Protein Assay Kit was used to determine sample concentrations, according to the manufacturer’s instructions (Thermo Fisher Scientific). The lysates were subjected to SDS-PAGE gel electrophoresis, transferred into 0.45 μm PVDF membranes and blocked for 30 min at room temperature with 3% skim milk (Sigma-Aldrich), prior to overnight incubation with the following primary antibodies: anti-PFKFB3 (conc. 1:2000, Cell Signaling, Danvers, MA, USA) and anti-ACTIN (1:5000 Invitrogen, Waltham, MA, USA). After a 60 min incubation with their respectively HRP-conjugated secondary antibodies (Invitrogen), membranes were developed using the SuperSignal West Pico chemiluminescence substrate (Thermo Fischer Scientific). Immunoblots were quantified using ImageJ 1.43u Software (National Institutes of Health, Bethesda, MD, USA. https://imagej.nih.gov/ij/ (accessed on 27 February 2021), 1997–2016).

### 4.11. Migration Assay

HCT-116, HT-29 and HUVEC cells were seeded into μ-dish-35 mm low culture inserts according to the manufacturer’s instructions (ibidi Integrated BioDiagnostics, Munich, Germany). After 12 h, the silicon brackets were removed and cell migration was observed at 6, 18, 24, 30 and 42 h (HCT-116 and HT-29 cells) and 3, 6, 9 and 12 h (HUVECs) time points. Images were taken using a Leica High Speed EC3 camera (Leica) on a Leica DM IL inverted microscope (Leica). Analyses were carried out using ImageJ 1.43u Software (National Institutes of Health, Bethesda, Maryland, USA. https://imagej.nih.gov/ij/ (accessed on 27 February 2021), 1997–2016) and GraphPad Prism™ 8 (GraphPad Software, Inc., La Jolla, CA, USA). The assay was performed three times.

### 4.12. Real-Time PCR Analysis

Total RNA was extracted using the RNeasy Mini Kit (Qiagen, Hilden, Germany) according to the manufacturer’s instructions. RT-PCR was performed using the SensiFAST SYBR^®^ No-ROX One-step Kit (Bioline, Memphis, TN, USA) on a BIO-RAD CFX384TM Real-Time PCR Detection System (BIO-RAD, Hercules, USA). Relative gene expression levels were quantified by using cyclophilin B as a housekeeping gene (2^[Ct Cyclophilin B − Ct target gene]^). Specific primer sequences are available upon request.

### 4.13. In Silico Analyses and Patients Survival Assessment

Tissue collection and gene expression profiling have been already described for the rectal cancer cohort [72,73,74]. Briefly, tissue samples were obtained from patients with locally advanced rectal cancer patients. Gene expression profiling was performed with human GE 4x44K version 2 Microarrays (Agilent Technologies, Santa Clara, CA, USA) in the context of the Clinical Research Unit 179 (KFO179). The survival analysis was performed using the R-package survival. The corresponding survival rates were computed by means of the Kaplan–Meier analysis and tested using the Cox proportional Hazards model. Disease-free survival was defined as the time from surgery until detection of locoregional or distant recurrence. All procedures were previously approved by the Ethics Committee of the University Medical Center Göttingen.

Regarding the colon cancer cohort, (The Cancer Genomic Atlas Network cohort) [20], *TCGAbiolinks* R-package was used to download transcriptome data of 519 samples (478 Primary solid Tumor samples and 41 Solid Tissue Normal samples) from the TCGA cohort on colon from the TCGA repository [75,76,77]. Data analysis was performed using R version 3.6.2. A trimmed mean of M-value (TMM)-normalization using R package *EdgeR* and VOOM-normalization was performed using R package limma before downstream analysis [78,79,80]. Survival analysis was performed using R-package survival which uses the Kaplan–Meier method to generate the survival functions, and the log-rank test to assess the significance of those functions. Data analysis was performed using the open-source software tool BIOMEX version 1.0-1 [81]. BIOMEX uses R-package survival which incorporates the Kaplan–Meier method to generate the survival functions, and the log-rank test to assess the significance of those functions. We used “Vital Status” as the event variable and “dead” as the event target and created an “overall survival variable” that is equal to “days to death” for dead patients, and to “days to last follow up” for the patients who are still alive for the survival analysis setting. Both the lower and upper quantile were set to 0.5 to define the groups to compare for overall survival.

### 4.14. Metabolomics

Metabolites extraction was achieved by a cold two-phase methanol–water–chloroform extraction as previously described [82,83]. Briefly, the samples were resuspended in 800 μL of precooled methanol/water (5/3) (*v*/*v*) followed by addition of 500 μL of precooled chloroform. Samples were vortexed for 10 min at 4 °C and then centrifuged (max. speed, 10 min, 4 °C). The methanol–water phase containing polar metabolites was separated and dried using a vacuum concentrator at 4 °C overnight and stored at −80 °C. For the detection of polar metabolites by LC–MS, a Dionex UltiMate 3000 LC System (Thermo Scientific) with a thermal autosampler set at 4 °C, coupled to a Q Exactive Orbitrap mass spectrometer (Thermo Scientific) was used for the separation of metabolites. Samples were resuspended in 70 µL of water and 10 µL of sample were injected, the separation of metabolites was achieved with a flow rate of 0.25 mL/min, at 40 °C, on a C18 column (Acquity UPLC HSS T3 1.8 μm 2.1 × 100 mm). A gradient was applied for 40 min (solvent A: 0 H_2_O, 10 mM tributyl-amine, 15 mM acetic acid—solvent B:Methanol) to separate the targeted metabolites (0 min: 0% B, 2 min: 0% B, 7 min: 37% B, 14 min: 41% B, 26 min: 100% B, 30 min: 100% B, 31 min: 0% B; 40 min: 0% B). The MS operated in negative full scan mode (*m*/*z* range: 70–1050 and 300–800 from 8 to 25 min) using a spray voltage of 4.9 kV, capillary temperature of 320 °C, sheath gas at 50.0, auxiliary gas at 10.0. Data was collected and analyzed using the Xcalibur software (Thermo Scientific).

### 4.15. Statistical Analysis

Data are presented as mean ± (Standard Error of the Mean) SEM. Statistical analysis was performed with GraphPad Prism™ 8 (GraphPad Software Inc.) using two-tailed Student’s *t*-test, unless otherwise indicated in the figure legends. *p* < 0.05 was considered statistically significant. * *p* < 0.05, ** *p* < 0.01 *** *p* < 0.001, **** *p* < 0.0001; ns, *p* > 0.05.

## 5. Conclusions

The development of new therapeutic drugs which could enhance patients’ response to treatment is a challenge in basic, translational and clinical research. The newly developed KAN0438757 showed to be effective in reducing glycolysis in colorectal cancer cells and their survival capabilities. Additionally, in vivo evaluation of KAN0438757 administration in mice showed to be well tolerated, without any signs of excessive toxicity. Therefore, inhibition of glycolysis by PFKFB3 targeting through KAN0438757 could be a promising approach in novel future therapeutic strategies.

## Data Availability

No new data were created or analyzed in this study. Data sharing is not applicable to this article.

## Supplementary Materials

The following are available online at https://www.mdpi.com/2072-6694/13/5/1011/s1, Figure S1: (A) LC-MS metabolomic analysis for HCT-116 cancer cells and HUVECs after 24 h treatment with KAN0438757. (B) RT-PCR analysis for migration-related genes performed with HUVEC cells after 12 and 48h treatment with KAN0438757, Figure S2: (A,B) RT-PCR analysis for migration-related genes performed with HCT-116 and HT-29 cancer cells after 12, 24 and 48 h treatment with KAN0438757, Figure S3: Additional experimental repetitions of Seahorse analysis performed with HCT-116, HUVEC, HT-29 and SW-1463 cells after 6h treatment with KAN0438757, Figure S4: (A–D) Additional experimental repetitions of RT-PCR analysis for migration-related genes performed with HUVEC cells after 12, 24 and 48 h treatment with KAN0438757.
