# Supplementary material for: Effects of the Novel PFKFB3 Inhibitor KAN0438757 on Colorectal Cancer Cells and Its Systemic Toxicity Evaluation In Vivo
Source: Cancers (Basel). 2021 Feb 28;13(5):1011. doi: 10.3390/cancers13051011 (PMC7957803; doi:10.3390/cancers13051011)
Supplement: Supplementary file 1 [file cancers-13-01011-s001.pdf]

cancers

MDPI

*Supplementary Materials*

# Effects of the Novel PFKFB3 Inhibitor KAN0438757 on Colorectal Cancer Cells and Its Systemic Toxicity Evaluation In Vivo

**Tiago De Oliveira, Tina Goldhardt, Marcus Edelmann, Torben Rogge, Karsten Rauch, Nikola Dobrinov Kyuchukov, Kerstin Menck, Annalen Bleckmann, Joanna Kalucka, Shawez Khan, Jochen Gaedcke, Martin Haubrock, Tim Beissbarth, Hanibal Bohnenberger, Mélanie Planque, Sarah-Maria Fendt, Lutz Ackermann, Michael Ghadimi and Lena-Christin Conradi**

Supplementary Figure 1

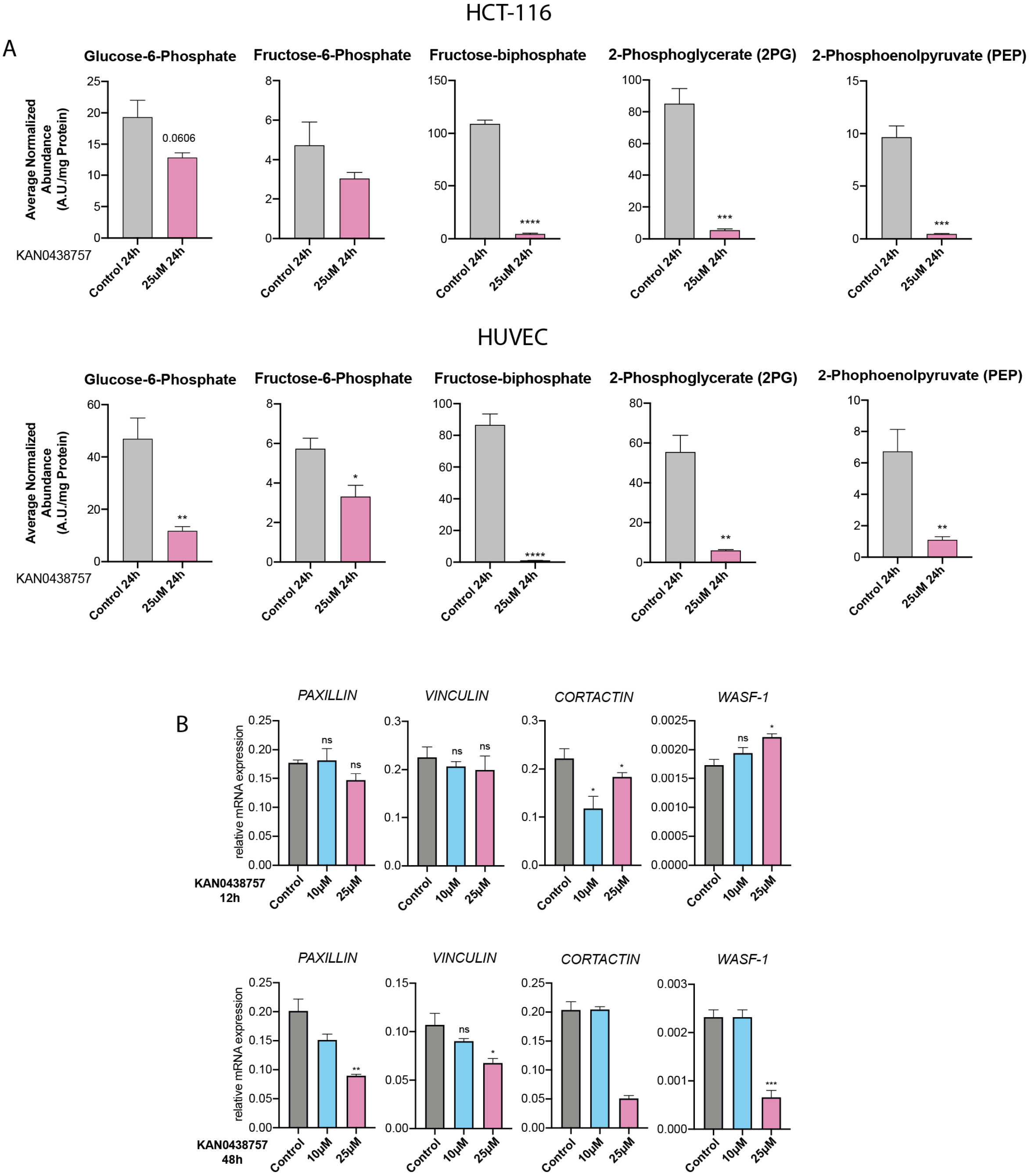

**Figure S1.** (**A**) LC-MS metabolomic analysis for HCT-116 cancer cells and HUVECs after 24h treatment with KAN0438757. (**B**) RT-PCR analysis for migration-related genes performed with HUVEC cells after 12 and 48h treatment with KAN0438757.

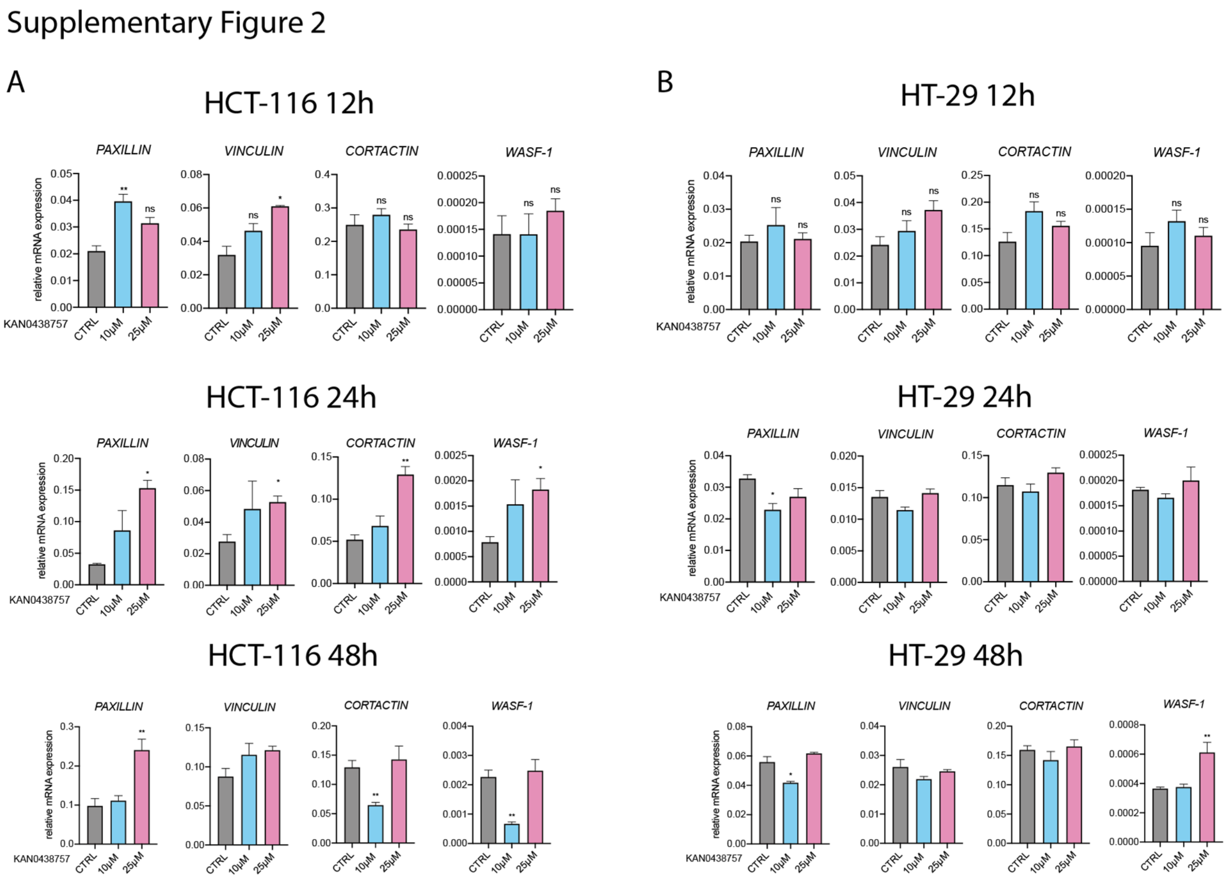


**Figure S2.** (**A**,**B**) RT-PCR analysis for migration-related genes performed with HCT-116 and HT-29 cancer cells after 12, 24 and 48h treatment with KAN0438757.

# Supplementary Figure 3

## Seahorse Analysis. Experimental Repetitions 2 and 3.

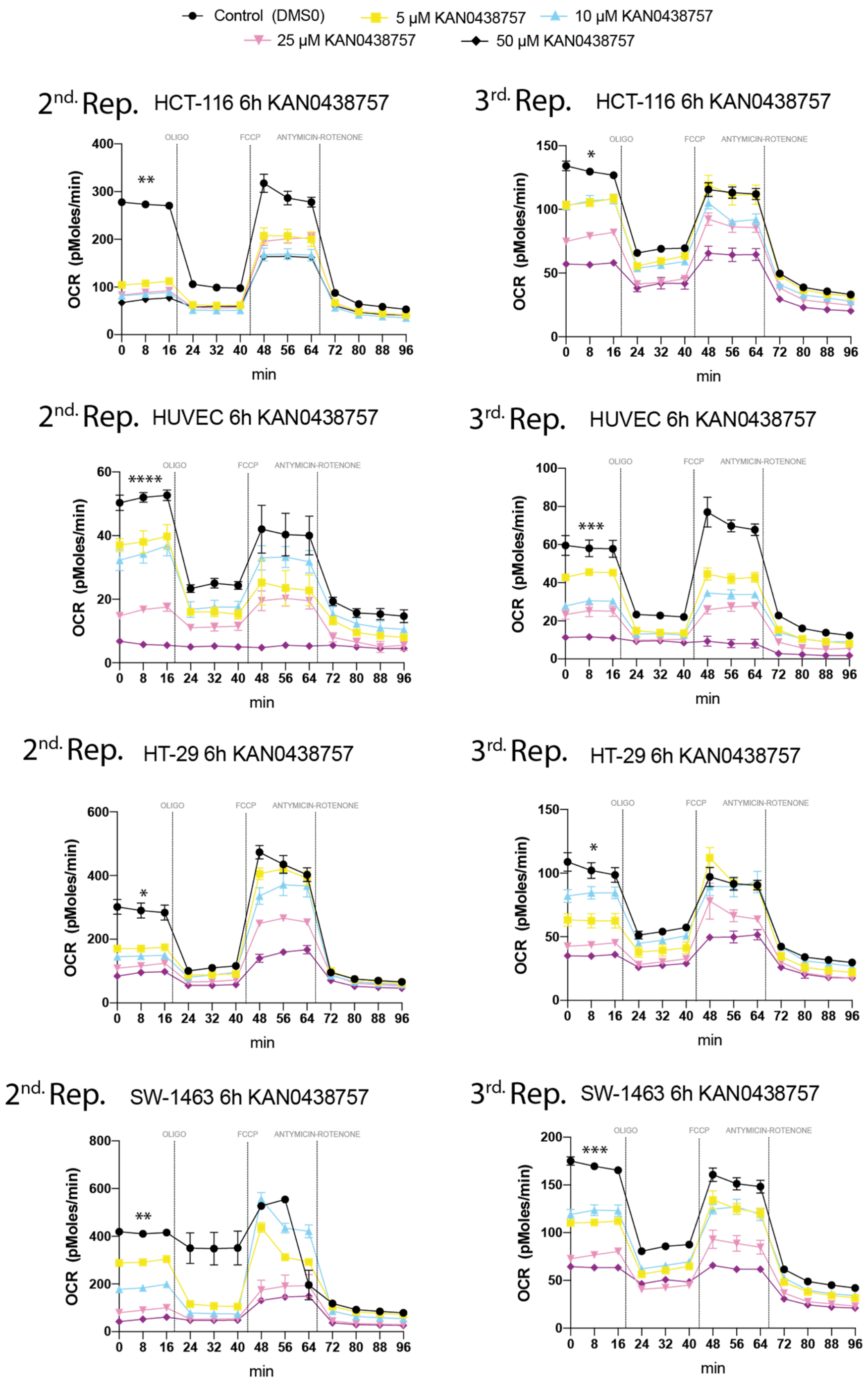


**Figure S3.** Additional experimental repetitions of Seahorse analysis performed with HCT-116, HUVEC, HT-29 and SW-1463 cells after 6h treatment with KAN0438757.

## Supplementary Figure 4

RT-PCR Analysis - migration and cytoskeleton-related genes. Experimental Repetitions 2 and 3.

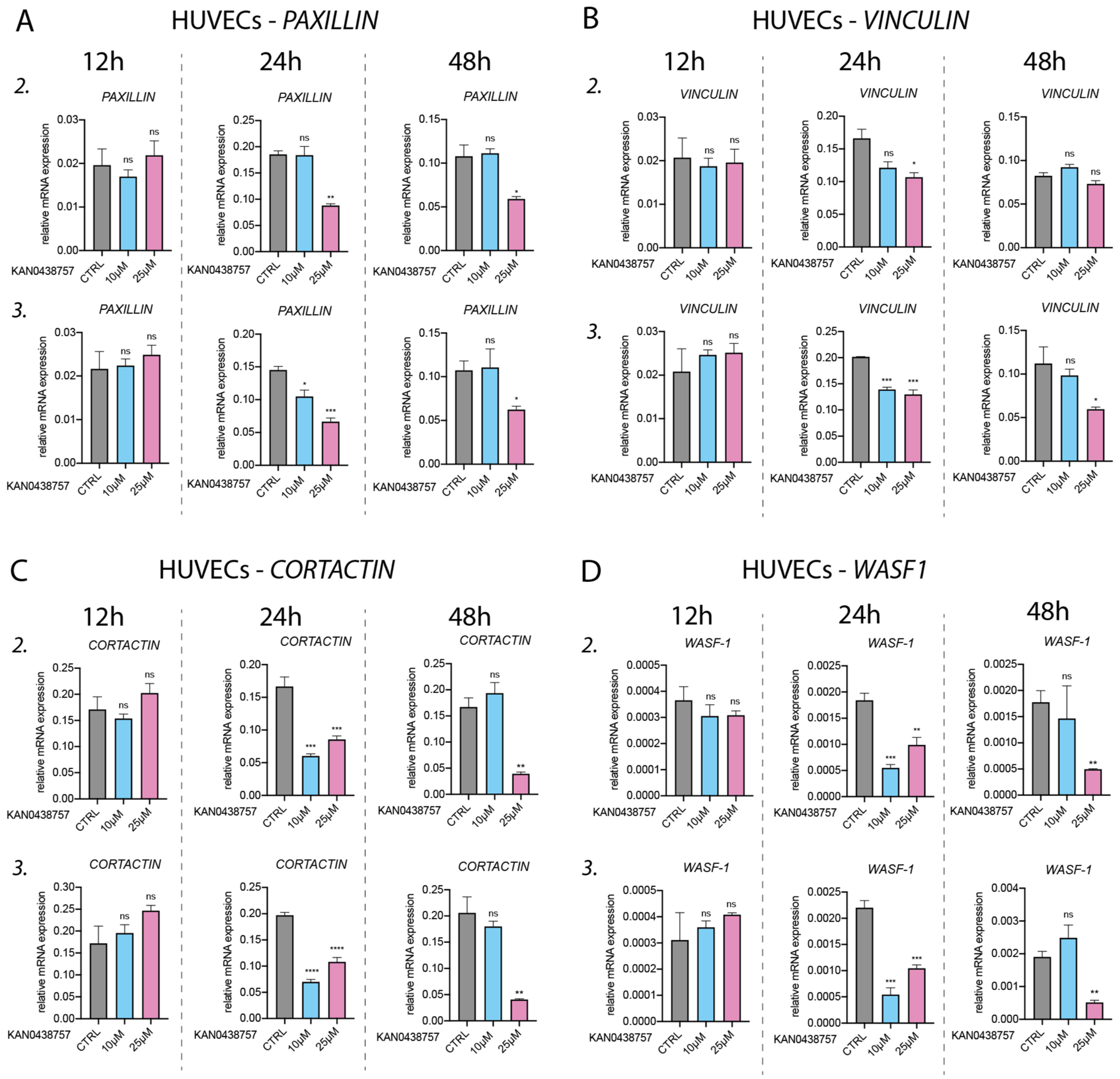


**Figure S4. (A-D)** Additional experimental repetitions of RT-PCR analysis for migration-related genes performed with HUVEC cells after 12, 24 and 48h treatment with KAN0438757.
